# Age and practice effects on inter-manual performance asymmetry

**DOI:** 10.3389/fpsyg.2014.01585

**Published:** 2015-01-15

**Authors:** Karen L. Francis, Priscilla G. MacRae, Waneen W. Spirduso, Tim Eakin

**Affiliations:** ^1^Motor Behavior Laboratory, Department of Kinesiology, University of San FranciscoSan Francisco, CA, USA; ^2^Motor Behavior Laboratory, Department of Sports Medicine, Pepperdine UniversityMalibu, CA, USA; ^3^Motor Behavior Laboratory, Department of Kinesiology and Health Education, The University of Texas at AustinAustin, TX, USA

**Keywords:** manual asymmetry, force control, aging, inter-manual performance asymmetry, HAROLD

## Abstract

Manual dexterity declines with increasing age, however, the way in which inter-manual asymmetry responds to aging is unclear. Our purpose was to determine the effect of age and practice on inter-manual performance asymmetry in an isometric force pinch line tracing task that varied in difficulty within segments. Thirty right-handed participants, five males and five females in each of three age groups, young (Y20), young–old (O70), and old–old (O80), practiced an isometric force pinch task for 10 trials with each hand on each of five consecutive days. Inter-manual performance asymmetry of the right and left hands was analyzed with a repeated measures analysis of variance (ANOVA) of asymmetry with age groups, practice, task difficulty, and hand as factors. The within-individual magnitude of asymmetry was also analyzed with a repeated measures ANOVA of manual asymmetry calculated as an asymmetry index (AI). *Post hoc* pair-wise comparisons were performed when significance was found. We observed no inter-manual performance asymmetry on this isometric tracing task among any of the age groups, either in the hand performance differences or in the magnitude of the AI. Age and practice interacted in terms of manual performance: the Y20 and O70 group improved *accuracy and task time* across the 5 days of practice but the O80 group did not. However, practice did not differentially affect the AI for *accuracy or task time* for any group. Accuracy of performance of the two hands was differentially affected by practice. All age groups exhibited poorer performance and larger AIs on the most difficult segments of the task (3 and 6) and this did not change with practice.

## INTRODUCTION

Inter-manual asymmetry, the commonly observed phenomenon that most humans use their right hand to execute high precision motor tasks, has been reported to develop throughout childhood and peak when young and middle-aged adults reach their highest level of skill (Raw et al., 2012; Gooderham and Bryden, 2013). Manual dexterity of both hands deteriorates with aging due to changes in neuromuscular structure and function as well as age-related declines in hand use and general physical activity (Carmeli et al., 2003; Ward and Frackowiak, 2003; Kalisch et al., 2006). Teixeira (2008) categorized manual tasks into three profiles: those associated with an asymmetrical right hand advantage (handwriting, aiming throwing, and maximal grip strength); more symmetrically performed tasks (anticipatory timing, grasping moving objects, and twisting and drilling performance); and tasks associated with asymmetrical left hand advantage (hand posture tasks). However, the effects of aging on inter-manual performance asymmetry has not been resolved, although most agree that age effects on inter-manual performance asymmetry are task-specific (Provins, 1997; Seidler, 2007; Teixeira and Teixeira, 2007; Raw et al., 2012; Saucedo Marquez et al., 2013).

Some studies found that older adults demonstrate less manual asymmetry than young adults, especially on tasks that have been observed to be highly lateralized (Teixeira, 2008). Examples are button pressing (Mattay et al., 2002; Rowe et al., 2006) manually tracing lines (Raw et al., 2012) and reaching tasks (Przybyla et al., 2011). Two models have been proposed to explain hemispheric asymmetry changes with age: the hemispheric asymmetry reduction in older adults model (HAROLD; Cabeza, 2002) and the right hemisphere aging model (Dolcos et al., 2002). According to the HAROLD model, prefrontal cortex activity tends to be less lateralized in older adults when compared to young adults as seen in cognitive tasks where older adults tend to show more bilateral activations than young adults (Cabeza, 2002). Przybyla et al. (2011) applied the HAROLD model to motor performance and found a reduction in manual asymmetries in older adults performing a horizontal plane reaching task. The right hemisphere aging model suggests that age-related cognitive declines affect functions located in the right hemisphere more than functions located in the left hemisphere (Dolcos et al., 2002). Weller and Latimer-Sayer (1985) found support for this model using a simpler task, the peg-board task, where older adults showed greater decline in left hand performance (hence right cortex function) than in right hand performance.

In addition to comparing performance between the right and left hand, researchers have measured inter-manual performance asymmetry by calculating the *differences* between the hands; that is, the extent of asymmetry within each person which can be measured as a within-person score or asymmetry index (AI) metric. A few investigators, using AI as a measure of the magnitude of difference between participants’ two hands’ performances, have found that older adults are more asymmetrical when compared to young adults on certain tasks, such as grasp control (Chua et al., 1995), graphic tracing (Francis and Spirduso, 2000), and graphic drawing (Teixeira, 2008). However, Teixeira (2008) also reported that these same older adults were less asymmetrical than younger adults when hand grip strength was assessed. Therefore, more research is needed to clarify age effects on inter-manual performance asymmetry.

Several researchers have analyzed the effects of practice on manual asymmetry. Asymmetries observed in young adults on movement tasks have been shown to be dramatically changed with task-specific practice (Peters, 1976; Perelle et al., 1981; Bryden and Allard, 1998; Teixeira and Teixeira, 2007) and these changes even generalized to a different but similar motor task (Teixeira and Okazaki, 2007). In several studies of young adults performing movement tasks, the non-preferred hand benefitted more from practice and thus inter-manual performance asymmetry was decreased in highly lateralized tasks such as drawing shapes (Halsband, 1992) and reverse printing (Parlow and Kinsbourne, 1989). Conversely, Perelle et al. (1981), who provided 5 days of practice on a manipulative dexterity test, found that both hands improved similarly. Practice has been shown to reveal age-related reductions in inter-manual performance asymmetry in transfer of training studies, in which benefits derived from practice of one limb were not equally transferred to the other limb. Inter-limb transfer of trajectory direction information for a reaching task occurred only from the non-dominant to the dominant arm for young adults, whereas final position information transferred in both directions in older adults (Wang et al., 2011; Pan and Van Gemmert, 2013).

The characteristics, profiles, and proposed mechanisms of inter-manual performance asymmetry in young and old adults, discussed above, have focused on coordinated movement tasks that require not only central planning and execution but also substantial information processing of neuromuscular-generated feedback during the movement. Relatively few studies of the effects of aging on possible inter-manual performance asymmetry of dynamic force control have been conducted, and even fewer are available regarding the effect of age and practice on these asymmetries. Two studies, both using isometric force control to move a computer cursor to screen targets, have shown that differences between young and old can be reduced to non-significance with practice. Christou et al. (2007) reported that after just 35 trials of practice with the left (non-preferred) hand, no age differences remained groups on endpoint force accuracy, although the age groups still differed in the adjustments made in motor-output variability and muscle activity associated with the initial improvements. Poston et al. (2008), also found that the left hand of older adults exhibited greater errors than those of young on the first day of practice, but these differences were eliminated by two additional days of practice, whether the practice was with the right or the left hand. Conversely, several other investigators, providing multiple trials over more than 1 day showed that older adults improved dynamic isometric force control tracing and tracking when they performed and practiced with one hand, but not as much as young adults improved (Spirduso and Choi, 1993; Lazarus and Haynes, 1997; Voelcker-Rehage and Alberts, 2005; Francis et al., 2012). Lazarus and Haynes (1997) found no age differences in the transfer of information from one hand to the other in a transfer of training paradigm requiring participants to track isometrically a randomly generated template. For all age groups, whichever hand practiced second made fewer errors, benefitting from the previously practiced contralateral hand.

In this study we examined the interaction of age and practice on inter-manual performance asymmetry of unilateral isometric force control for each hand, particularly with regard to whether older adults are less asymmetrical than young adults, and whether the magnitude of inter-manual performance AI is lower in older adults. We also examined the interaction of age and practice, to determine what effect 5 days of practice has on inter-manual performance asymmetry and AI. Finally, because it is well-documented that age differences in motor task performance increases as task difficulty increases (Voelcker-Rehage, 2008), we used a force control template shown to have two segments requiring greater control than the other four, to determine whether inter-manual asymmetries and AI are influenced by age and practice and task difficulty.

## MATERIALS AND METHODS

### PARTICIPANTS

Thirty participants, five males and five females in each of three age groups: Y2 (mean age = 21.4 years, SD = 3.6, age range: 18–23 years), O70 (mean age = 69.7 years, SD = 2.6, age range: 65–74), and O80 (mean age = 78.5 years, SD = 2.8, age range: 75–84). The age range for the two older age groups were chosen because 65–74 is often referred to as the “young–old” while the 75–84 is termed the “old–old” (Spirduso et al., 2005b). The young adults were undergraduate volunteers from a university. The older adults were recruited from the local community and all had completed some college (mean years of school = 16.2 years, SD = 2.6). All participants were right-handed according to the Edinburgh handedness inventory (minimum score for right hand dominance = +0.90; Oldfield, 1971), with normal to corrected vision, no diagnosed cognitive or neurological disorders, free of severe arthritis, could ambulate unassisted, and lived independently with no prior experience with the apparatus. Participants gave informed consent (approved by two university IRB boards) affirming their willingness to participate in this research study.

### INSTRUMENTATION

The manual force quantification system (MFQS) was designed to quantify low levels of isometric force control in a dynamic pinch task that requires modulation of inter-digit forces (Spirduso et al., 2005a). The instrumentation quantified the amount of force applied to each of a pair of transducers by individual digits, either the thumb or index finger of the (preferred) right hand or those of the (non-preferred) left hand. The amount of force produced by each digit was manifested directly as the position of a cursor on a computer screen such that one force transducer controlled the cursor movement parallel to a horizontal x-axis, and a second force transducer controlled the cursor movement parallel to a vertical y-axis.

**FIGURE 1:**
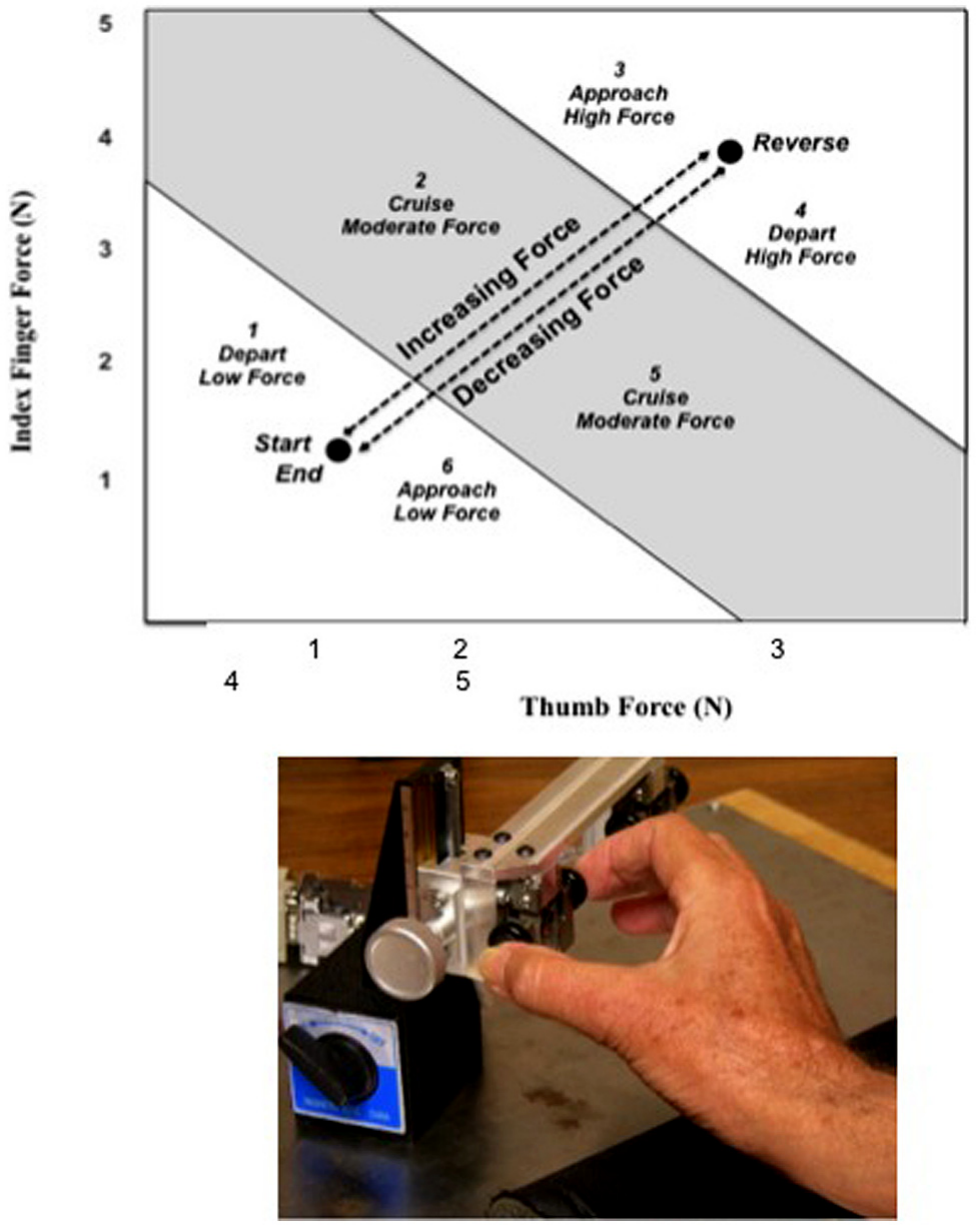
**Force tracing template parsed by segment categories.** The lower black filled circles indicate both the *Start* and *End* circles and the upper black filled circle indicates the *Reverse* circle. The *Start* circle is associated with Segment 1 (depart, increasing force); the *Reverse* circle is associated with both Segment 3 (approach, increasing force) and Segment 4 (depart, decreasing force); and the *End* circle is associated with Segment 6 (approach, decreasing force). Segment 2 (cruise, increasing force) and Segment 5 (cruise, decreasing force) are not associated with circles. The segment locations and numbers are not visible to the participant, but are developed post-data collection for statistical analyses.

A 45° tracing template, representing equal forces from each digit, was projected onto a monitor screen and included the target line connected by *Start, Reverse,* and* End* circles (**Figure 1**). Tracing in this task required the participant to begin and end the task at circles located respectively at the identical position on the computer screen. The participant had a full view of the current position of the cursor, but was provided no displayed retention of the cursor’s cumulative tracing trajectory. The first part of the task required a net force application to move the cursor from the *Start* to the *Reverse* circle. The second part of the task required a net decrease in force from the *Reverse* to the *End* circle. Each of the circles lit up and a beep was heard when the cursor first contacted its own radius of acceptance, 0.098 newtons (N) in each instance.

The MFQS apparatus was comprised of two strain gages mounted to a base on a platform that was positioned and locked into place for each participant. The heel of the hand was anchored on the console and remained in contact with the console throughout the trial. The range of each strain gage was 0 to 4.45 N and the non-linearity of each was less than 1%. Force from the thumb controlled the cursor position on the horizontal x-axis and force from the index finger controlled the position on the vertical y-axis. Thus, simultaneous force of equal magnitude exerted by both digits kept the cursor on the 45° tracing line template with perfect geometric accuracy. This instrumentation also allowed for independent isometric force measurements by either digit. In this study, because the highest level of force required by the task at the return circle was only 7 N (simultaneous application of 3.5 N with the thumb and 3.5 N with the index finger), neither strength nor fatigue was a confounding factor. According to Herring-Marler et al. (2014) the average maximum pinch strength (combined thumb and finger forces) for older adult women and men was ∼49 and 71 N respectively. Therefore the upper boundary of the high, middle, and low force levels of this task were 11, 8.4, and 5.8% of their mean maxima.

At each sampling instant (200 Hz) the instrumentation automatically recorded the thumb and finger force values along with a numerical value assigned to those data pairs which identified the current force level of the task in terms of increasing or decreasing force modulation. The software provided continuous timed data collection during the course of a trial and a visual display showing the cursor position to the participants. The data acquisition for this experiment utilized a virtual instrument (VI) application constructed with LabVIEW (National Instruments). Raw data for each trial were collected as a time series of horizontal and vertical cursor coordinate positions. Values were converted from grams to the corresponding force amplitude values in newtons (N). Trials that revealed a lapse or discontinuity in performance, such as intermittent gaps in which the release of contact with one or both strain gages was apparent, were discarded.

The target line was spatially divided into six equal task segments as shown in **Figure 1**. The segments were categorized by their proximity to a target: *Start*, *Reverse*, and *End* circles. The segments could also be distinguished by force level: low (5.8%), middle (8.4%), and high (11.0%). Thus each segment can be defined as follows: (1) *departing Start* circle, increasing force, low force level; (2) *cruising*, increasing force, middle force level; (3) *approaching* Reversal circle, increasing force, high force level; (4) *departing* Reversal circle, decreasing force, high force level; (5) *cruising*, decreasing force, middle force; and (6) *approaching End* circle, decreasing force, low force. The segmentation was created *post hoc* during the analysis and participants had no knowledge or visual indication of these categories. These segments were a factor that was included in the analysis, but they were not visible and were thus unknown to the participants. Our previous research has consistently shown that more errors are made and more time taken to approach the target or to reverse force direction (Spirduso et al., 2005b; Griffin et al., 2009; Eakin et al., 2012; Francis et al., 2012; Herring-Marler et al., 2014).

### PROCEDURES

After giving consent, the participant was seated directly in front of the computer monitor, with the index finger and thumb of the right or left hand (hand order counterbalanced) resting on the console transducers and the arm bent at the elbow, forearm in a sagittal plane. To enhance consistency of performance and eliminate any confounding by wrist flexion, the participant kept the ulnar border of the hand anchored on the console box at all times. The thumb was positioned on the transducer nearest the participant and the index finger was positioned on the other strain gage button. Fingers not being used for contact with the strain gages were kept in a static and comfortably flexed position with no force exertion against any surface. The goal of the task was to coordinate the forces between the thumb and index finger by increasing or decreasing pressure to the transducers in such a way that the cursor progressed along the target line on the computer monitor from one circle to another (**Figure 1**).

Each participant completed an initial screening test that involved joystick control, before beginning the MFQS trials. The screening test used the same tracing template as the MFQS task except that the cursor was manipulated by single-hand positioning of a joystick which controlled the cursor on both x and y axes simultaneously. This screening task was intuitive and was easily accomplished by all participants, but because the testing set-up, task goals, and templates were exactly the same as in the force control task, it provided an efficient assessment of the participants’ basic comprehension of the force control task instructions and instilled confidence in his/her ability to execute the task. This screening task also provided an indication of whether visual-spatial problems existed that would confound the assessment of force control.

Participants were tested five consecutive days and each testing session lasted ∼1 h. Each of the 5 days of practice followed the same protocol, except for the first day, which included signing the consent form, listening to the instructions, and performing two joystick trials with the right and left hands. This was followed by two practice trials with the right and left hands on the tracing task, and then 10 MFQS tracing trials for each hand. Hand order was counterbalanced across days. On each of the following 4 days, 10 trials were completed for the right and left hands. Participants were instructed “to complete the task as fast and accurately as possible.” Accuracy was based on the ability to maintain contact with the tracing line throughout the tracing.

### STATISTICAL ANALYSES

In order to examine inter-manual asymmetry between-hands and within-person and we conducted two repeated measures analysis of variances (ANOVAs). The first analysis was a mixed model ANOVA designed to determine if there were performance differences between the right and left hand that were related to the independent variables of Age (Y20, O70, O80), Days (1–5), and Segments (1–6) for Time and root mean square error (RMSE). *Post hoc* pair-wise comparisons were performed when significant results were found. The dependent measures for this analysis were the (1) natural logarithm of time in seconds required to traverse the entire task from *Start* circle to *End* circle and (2) accuracy, inferred from directly measured RMSE. The RMSE of an individual trial was determined by the collective individual error magnitudes from each sampling instance. That individual sampling error was the shortest distance (perpendicular line) from the cursor position to the template line because the error could not be allocated separately to the two digits if the target were a line rather than a single position.

Thus the individual error for a particular sampling *i* was

$$e_{i}=\frac{x_{i}-y_{i}}{\sqrt{2}}$$

and RMSE was defined as

$$RMSE=\sqrt{\frac{\Sigma_{i=1}^{N}e_{i}^{2}}{N}}$$

This indirect measure of accuracy represents the deviation from a perfect performance which would require a participant to increase and decrease force equally between the thumb and index finger, thus moving the cursor directly on the line between circles. The greater the distance between forces contributed by each digit at any sampling instance, the higher will be the contribution to the RMSE score and the less accurate will be the performance relative to the goal of the task.

A second ANOVA was performed on the absolute AI, in which the between-group factor was Age and the repeated measures were Days (1–5) and Segment (1–6). This analysis was designed to determine if there were significant differences in the magnitude of within-person manual asymmetry for Time and RMSE. *Post hoc* pair-wise comparisons were performed when significant results were found. The dependent measures for this analysis were the absolute AI for Time and RMSE. An AI was derived for each segment of each paired right and left hand trial sequence position on each practice day for each participant, based on the method of Teixeira and Teixeira (2007). The pairings were temporal such that the first right hand trial of a particular day was paired with the first left hand trial of that day, and so forth. If *R_ij_* represents a variable score for segment *j* of right hand trial *i* and *L_ij_* represents the score for segment *j* of the paired left hand trial, then the asymmetry magnitude for the paired segment was

$$A_{ij}=\left|\frac{R_{ij}-L_{ij}}{R_{ij}+R_{ij}}\right|$$

and the AI dependent variable for segment *j*, reflecting *A_ij_* values for that segment over *N* trial pairs, was

$$AI_{j}=\overset{N}{\underset{i=1}{\Sigma }}\frac{A_{ij}}{N}$$

where, *N* = 10 for sets with no discarded performances. Pair-wise comparisons were performed when further *post hoc* analyses were indicated.

The data for 30 participants were initially organized by trial for a total of 3000 trials, or 20 trials (10 for the right and 10 for the left hand) on each of 5 days. Six scores for each dependent variable were produced for each trial because the task contained six segments. Trials that did not meet the pre-established criteria were removed from further analysis. Because the missing trials were few (<1% of data) and scattered among participants, testing for bias was unnecessary. The Estimation–Maximization technique was used to replace missing values using the mean for participant’s trials on a given day. The time scores did not meet the criteria for assumption of a normal distribution. However, the natural logarithms of the time scores did meet such criteria, so that transformed time scores were used for statistical significance evaluations. To keep interpretation of results on a more conceptual level, references to and percentage changes in the Time variable are given with respect to directly measured time intervals, not to the natural logarithms of those values. However, all *p* values presented in conjunction with the Time variable are those obtained from statistical analysis using the natural logarithm values. All interpretations of statistical significance or non-significance involving directly measured time scores are made with the assumption that any relationship involving the mean of the log of time scores will also hold for the corresponding relationship involving the arithmetic mean of direct time scores.

## RESULTS

### EFFECT OF AGE AND HAND ON FORCE CONTROL

Age as a main effect averaged over all other independent variables was significant for both Time [*F*(2,27) = 4.04, *p* < 0.05; η^2^ = 0.23] and RMSE [*F*(2,27) = 2.72, *p* < 0.05; η^2^ = 0.28]. As revealed in the *post hoc* comparisons the Y20 group was faster than the O80 group (*p* < 0.05) and more accurate than the O70 (*p* < 0.05) and O80 groups (*p* < 0.05).

**FIGURE 2:**
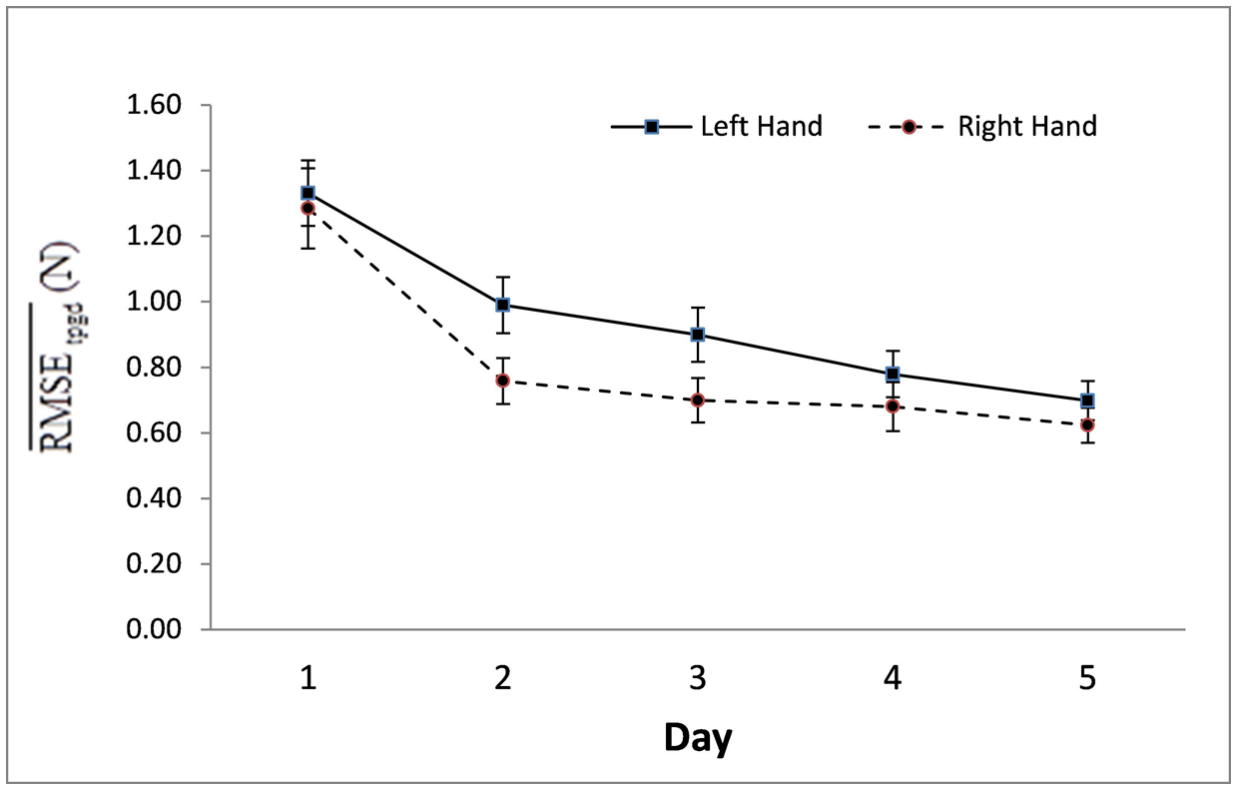
**Mean RMSE and standard errors of right and left hand performances by practice day as averaged over all segments (s) of all respective performance hand trials (t) of all participants (p) of all age groups (g).** The mean RMSE scores of right vs. left hand performance were significantly different on Days 2 and 3 (*p* < 0.05).

**FIGURE 3:**
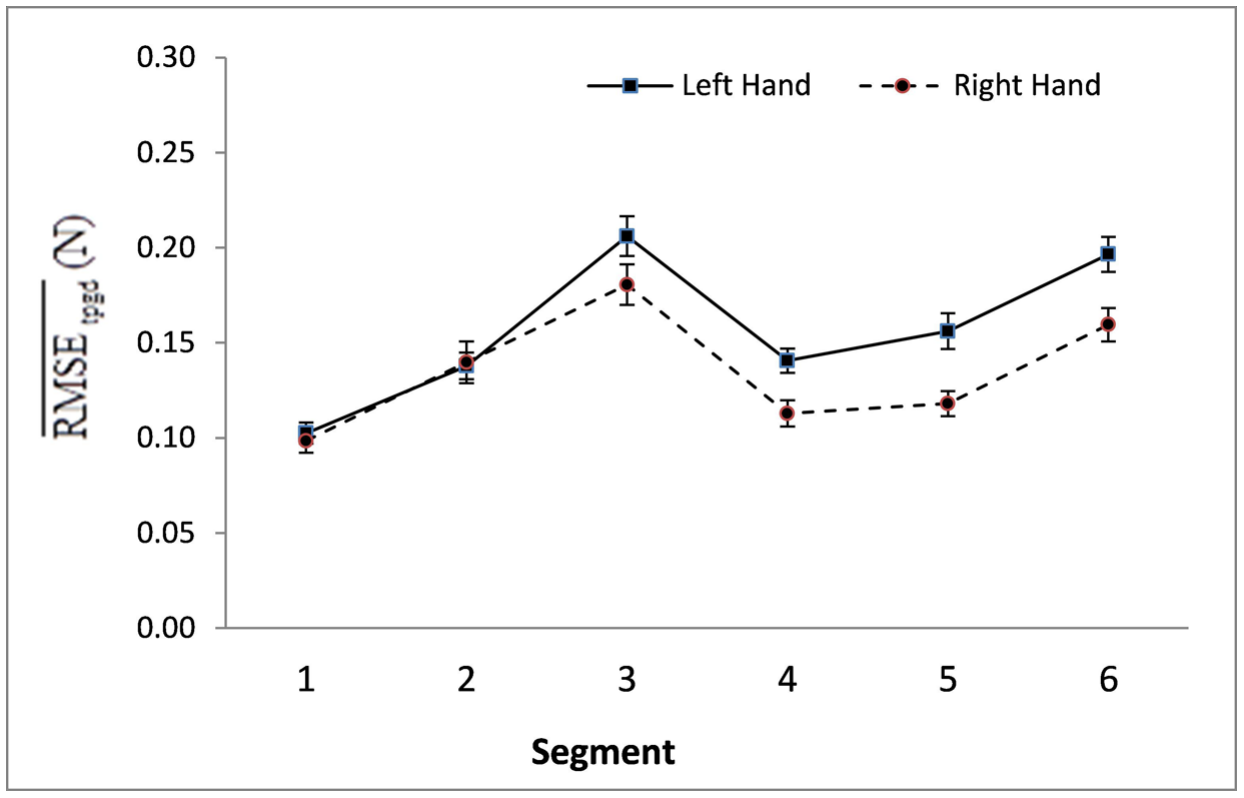
**Mean RMSE and standard errors of right and left hand performances by practice day as averaged over all segments (s) of all respective performance hand trials (t) of all participants (p) of all age groups (g).** The mean RMSE scores of right vs. left hand performance were significantly different on Days 2 and 3 (*p* < 0.05).

These participants, young and old, did not exhibit a between-hand performance difference for time taken to complete the task. However, the Hand × Day interaction for RMSE was significant [*F*(4,24) = 3.99, *p* < 0.05; η^2^ = 0.40]; with the right hand making lesser error, when compared to the left hand, on Days 2 and 3 (*p* < 0.05; **Figure 2**). The Hand × Segment interaction for RMSE was significant [*F*(5,23) = 5.94, *p* < 0.01; η^2^ = 0.56] with the right hand making lesser error, when compared to the left hand on Segments 3–6 (*p* < 0.05; **Figure 3**).

The three-way interaction of Hand × Segment × Age [*F*(10,46) = 3.27, *p* < 0.01; η^2^ = 0.42] for RMSE also was significant. The left hand of the Y20 demonstrated lesser error than the left hand of the O80 for Segments 1–4 (*p* < 0.05). Additionally, the right hand of the Y20 demonstrated fewer errors than the right hand of the O70 for Segment 3 (*p* < 0.05) and fewer errors than the right hand of the O80 for Segment 6 (*p* < 0.05).

### EFFECT OF PRACTICE ON FORCE CONTROL

**FIGURE 4:**
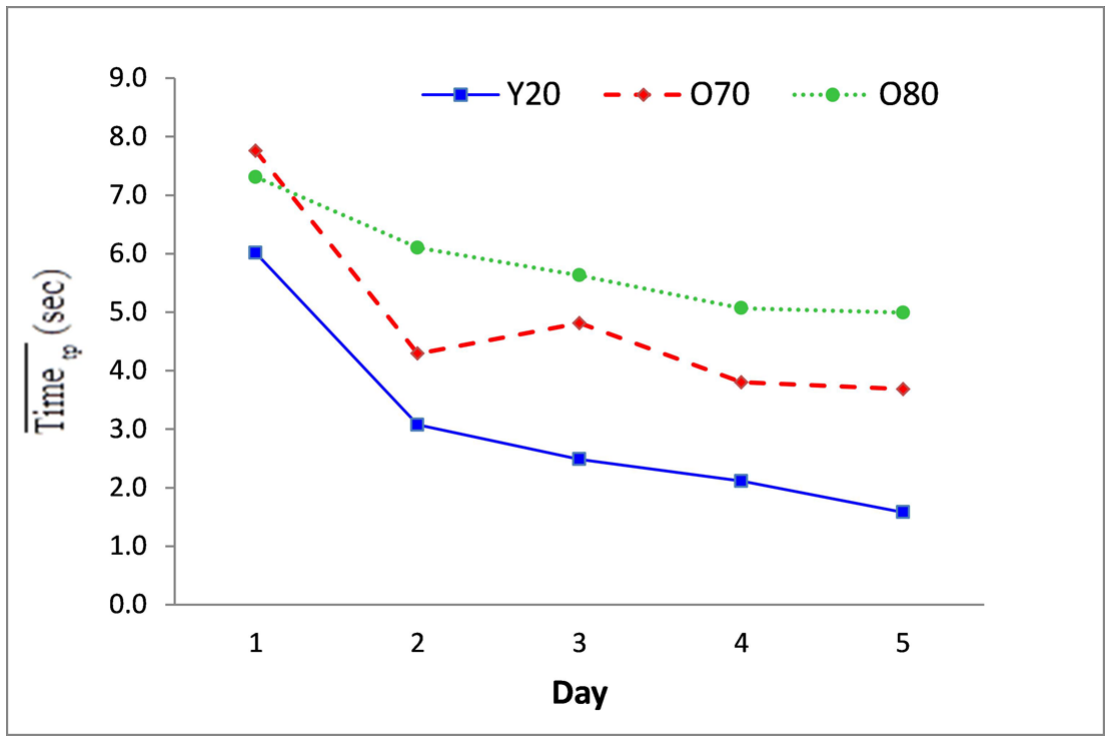
**Age group mean whole Task Time scores by practice day as averaged over all trials (t) of all participants (p).** There was a statistically significant difference between the arithmetic mean time score for the Y20 vs. O80 groups on Days 4 and 5 (*p* < 0.05).

The Day main effect for Time [*F*(4,24) = 32.60, *p* < 0.001; η^2^ = 0.85] and RMSE [*F*(4,24) = 16.07, *p* < 0.001; η^2^ = 0.65] were significant. As revealed in the *post hoc* comparisons, the time taken to complete the task was longer on Day 1 compared to all other days (*p* < 0.05). Participants made more error on Day 1, compared to all other days (*p* < 0.01) and on Days 2 and 3 compared to Day 5 (*p* < 0.05). Age interacted with Day for Time [*F*(8,48) = 3.44, *p* < 0.01; η^2^ = 0.37; **Figure 4**] such that the Y20 were faster than O80 only on Days 4 and 5 (*p* < 0.05).

### EFFECT OF SEGMENT ON FORCE CONTROL

**FIGURE 5:**
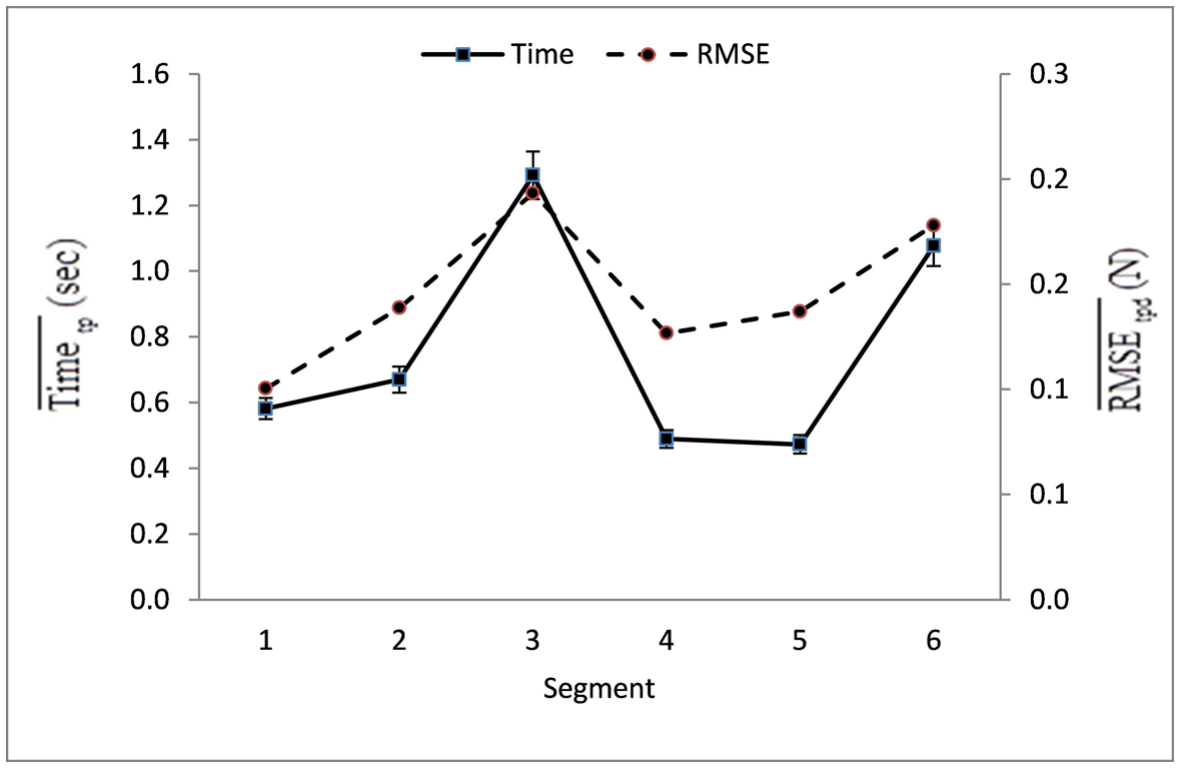
**Mean Task Time and standard errors by segment as averaged over all trials (t) of all participants (p) in all groups on all practice days (d).** The arithmetic mean time and the mean RMSE score for Segments 3 and 6 have a statistically large significant difference from those mean scores of all other segments (*p* < 0.01).

The Segment main effect was significant for both Time [*F*(5,23) = 31.96, *p* < 0.001; η^2^ = 0.87] and RMSE [*F*(5,23) = 19.49, *p* < 0.001; η^2^ = 0.81]. As revealed in the *post hoc* comparisons, participants performed slower and with more errors on segments requiring target contact or change in force direction (Segments 3 and 6) when compared to all other segments (*p* < 0.01; **Figure 5**).

### EFFECT OF AGE ON THE ASYMMETRY INDEX

Manual asymmetry for time taken to complete the task, averaged over days and segments, was not significantly different among the three age groups. Additionally, there was no Age effect on RMSE and no Day × Age interaction for manual asymmetry.

The Segment × Age interaction for manual asymmetry was not significant for Time or RMSE when performance was averaged across days. However the Day × Segment × Age interaction for RMSE was significant [*F*(40,16) = 2.63, *p* < 0.05; η^2^ = 0.86]. As revealed in *post hoc* comparisons, both the Y20 and the O70 groups were more asymmetric than the O80 group but only on Day 4, Segment 6 (*p* < 0.01 and *p* < 0.05, respectively).

### EFFECT OF PRACTICE ON THE ASYMMETRY INDEX

Practice resulted in a significant decrease in manual asymmetry of time taken to complete the task [*F*(4,24) = 2.77, *p* < 0.05; η^2^ = 0.32]. As revealed in *post hoc* comparisons, manual asymmetry for Time was lowest on Day 4 when compared to Days 1, 2, and 5 (*p* < 0.01). Practice had no significant effect on manual asymmetry of RMSE. In addition, the Day × Segment interaction was not significant for Time or RMSE.

### EFFECT OF SEGMENT ON THE ASYMMETRY INDEX

**FIGURE 6:**
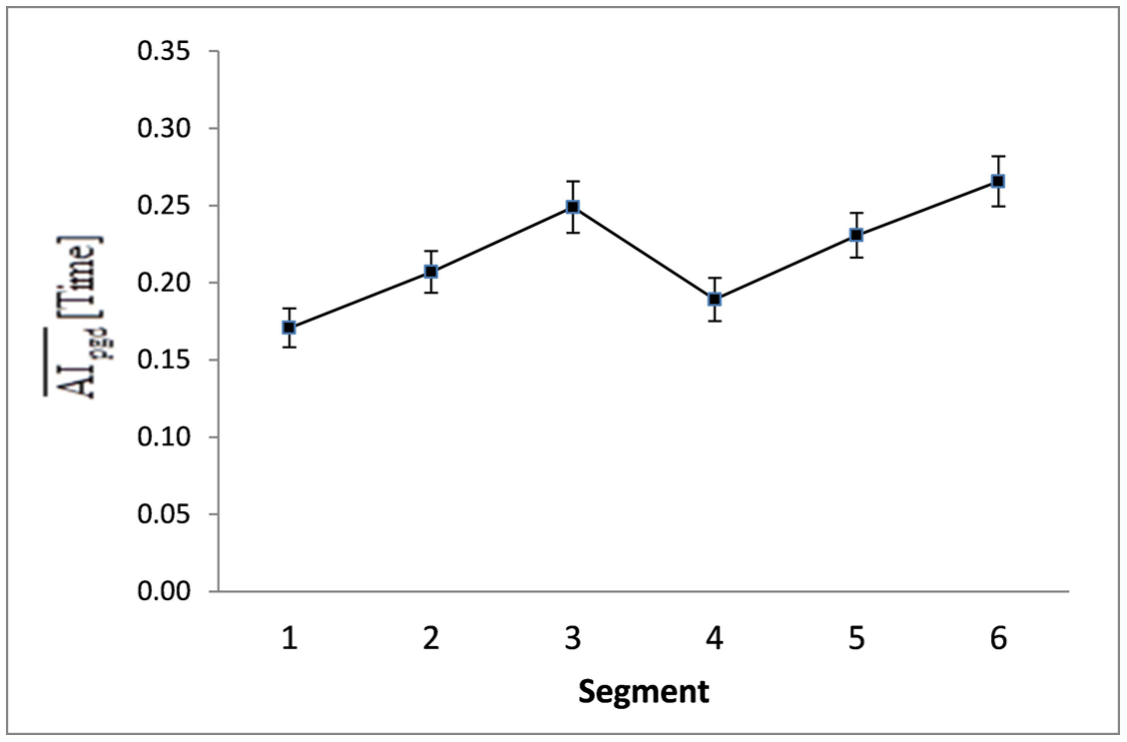
**Mean absolute asymmetry index and standard errors of the dependent variable *Time* for each of the six segments as averaged collectively over all participants (p) in every group (g) on every practice day (d).** The mean AI time scores were significantly different on Segments 3 and 6 (*p* < 0.05).

The Segment main effect for manual asymmetry of time taken to complete the task was significant [*F*(5,23) = 8.14, *p* < 0.001; η^2^ = 0.64]. As revealed in *post hoc* comparisons, manual asymmetry was greatest on Segments 3 and 6 (*p* < 0.01; **Figure 6**). Proximity to the reverse circle (Segment 3) and end (Segment 6) had a powerful effect on manual asymmetry for time taken to transverse the different segments. The magnitude of manual asymmetry for RMSE, expressed across segments, did not change with practice.

## DISCUSSION

Three main findings emerged from this study. First, we observed no inter-manual performance asymmetry on this isometric tracing task among any of the age groups, either in the hand performance differences or in the magnitude of the AI. Second, the Y20 and O70 group improved performance across the five days of practice although the O80 group did not and practice did not differentially affect the AI for the three age groups. Practice also differentially affected the accuracy of performance of the two hands. Third, all age groups exhibited poorer performance and larger AIs on the most difficult segments of the task (3 and 6) and this did not change with practice.

### AGE AND INTER-MANUAL PERFORMANCE ASYMMETRY

In this isometric force tracing task, there were no significant age differences in inter-manual performance asymmetry for time or accuracy, as evidenced by the lack of significant age interactions with any of the other study factors. If the HAROLD model were operative we would expect older adults to be less asymmetrical compared to young adults and if the Right Hemisphere Aging model were operative we would expect older adults to be more asymmetrical compared to young adults, even after five days of practice. The lack of age differences in asymmetry in this isometric tracing task are not explained by either model but may be related to the observations made by Paizis et al. (2014) who found age differences in inter-manual asymmetry that were based on whether the task required actual movements of the arms or imagined arm movements. Their older adults exhibited inter-manual asymmetry in actual pointing movements, but not during their imagined pointing movements. Thus, in both their study and the present study, inter-manual asymmetry was not seen when little or no movement occurred. Our findings suggest that planning, execution, and central processing of visuospatial feedback required for this isometric force tracing task were not negatively affected by age, even in the oldest age group. Our older adults showed no signs of age-related degradation of asymmetry. Our results provide yet another example that age changes in manual asymmetry are not global but rather, are task-specific (Chua et al., 1995; Francis and Spirduso, 2000; Seidler, 2007; Teixeira, 2008; Voelcker-Rehage, 2008). Isometric line tracing, which requires gradual increases and decreases at low force levels would fall in Teixeira’s (2008) manual asymmetry category he designated as *symmetric* performance, rather than *inconsistent* or *asymmetric* performance.

One explanation for the symmetry observed in this task may be that isometric force line tracing can also be categorized as a dynamic visuospatial task accomplished with the muscle activity held at a fixed length. Movement tasks such as reaching and hand drawing require concentric muscular contractions (muscle activity shortening) and eccentric contractions (muscle activity lengthening) and multiple joint angle changes, all of which would provide additional sensory feedback processing (Proske and Gandevia, 2012). Other investigators using dynamic isometric force control tasks such as matching different force levels (Harabst et al., 2000), force-tracking sine waves (Voelcker-Rehage and Alberts, 2005), and randomly shaped templates (Lazarus and Haynes, 1997) reported age differences in inter-manual performance asymmetry, but it is likely that the cortical planning and execution for these tasks were more complex.

### AGE AND PRACTICE EFFECTS ON INTER-MANUAL PERFORMANCE ASYMMETRY

Five days of practice decreased the amount of time taken to complete the task, but not similarly for all three groups, and the interaction of Age and Practice was significantly different only for time taken to complete the task, not for accuracy of performance (**Figure 4**). No age group differences were observed across the first 3 days of practice, but by Days 4 and 5 the young group completed the task significantly faster than the oldest group. In addition, both the Y20 and O70 were significantly faster than their own Time on Day 1 while the O80 group demonstrated no significant changes in time to complete the task across 5 days of practice. Our findings support the many studies that have shown that with practice both young and older adults improve in fine movement skills (e.g., Bock and Schneider, 2002; Kennedy and Raz, 2005; Rodrigue et al., 2005; Seidler, 2007; Voelcker-Rehage, 2008) and also in isometric force control tasks (Lazarus and Haynes, 1997; Voelcker-Rehage and Alberts, 2005; Poston et al., 2008; Camus et al., 2009; Sosnoff and Voudrie, 2009; Francis et al., 2012).

The largest practice-related changes in isometric tracing occurred in the O70 group from Days 1 to 2, a result that also occurred in two other studies: Poston et al. (2008) in an isometric force matching task and Voelcker-Rehage and Alberts (2005) in an isometric tracking study. However, our results do not agree with Christou et al. (2007) who reported that older adults approximately the same age as our O70 group performed a force matching task with time errors and endpoint accuracy errors similar to those of a young group after only 35 trials of practice. Our oldest group did not significantly decrease task time throughout 5 days of practice. Several other researchers of isometric force control have proposed that no matter how much older adults practiced, they could never close the age gap in tracking an irregular template pattern (Lazarus and Haynes, 1997; Voelcker-Rehage and Alberts, 2005), and index finger force matching (Sosnoff and Voudrie, 2009). These researchers suggested that older adults could not improve their processing of target-related sensorimotor feedback quickly enough, and could not reduce motor unit firing rate variability which has been shown to improve inter- and intra-muscle coordination (Laidlaw et al., 1999; Kamen and Knight, 2004; Griffin et al., 2009). Indeed, Pratt et al. (1994) provided evidence, and more recently Christou et al. (2007) revealed that in many cases even when older adults’ performances appear not to be different from those of young adults, the mechanisms underlying the movements of older adults can be qualitatively different. In their study of isometric index finger abduction time and accuracy, they suggested that the age differences in target accuracy that disappeared after 35 practice trials were associated with timing adaptations of the agonist and antagonist balance of electromyographic (EMG) activity. Young adults adjusted both the agonist and antagonist EMG to improve force endpoint accuracy, whereas old adults adjusted only the agonist muscle EMG to improve force endpoint accuracy.

Unlike the significant age group differences in time over the 5 days of practice, none of our age groups decreased errors across days. This result is in contrast to that of Voelcker-Rehage and Alberts (2005) study whose participants, approximately the same age (67–75) as our O70 age group, were significantly less accurate as indicated by their ability to stay within a target range. The differing results could be because the isometric force matching task in the Voelcker-Rehage and Alberts (2005) study was a more difficult task differing from our task in at least three ways number of force direction reversals (12 vs. 2), length of task (30 vs. 10 s), and range of template peak force levels (2–5 vs. 6–11%). All of these differences would increase the difficulty level of their task, and it is well-documented that increasing difficulty level increases age decrements in fine motor tasks (Spirduso et al., 2005b).

The magnitude of AI for time changed with 5 days of practice but not for accuracy. However this finding was attributable to Day 4 only and there was no trend or evidence that the participants’ hand performances were either systematically converging or diverging as a result of age or practice. Also, given the lack of a significant Age × Day interaction for error suggests that the change in AI was similar for the young and older groups across days.

Practice across 5 days had a differential effect on accuracy of the two hands. As revealed in the significant Hand × Day interaction for RMSE, the right and left hands, combined across age groups, erred in tracing the template erred in tracing the template almost identically on the first day, but the two hands diverged along different trajectory paths to arrive at almost identical mean error on the last day (**Figure 2**). The right hand of the combined age groups decreased mean error acutely from Day 1 to Day 2 and then plateaued, making almost no more decrease in mean error throughout the rest of the practice days. Conversely, the left hand mean error decreased more gradually, catching up to the right hand level of performance so that the two hands performed similarly on Day 4 and Day 5.

These results are partially consistent with those from studies of young adults in which right and left hands practiced anisometric (movement) tasks and the two hands that performed asymmetrically on initial trials converged to perform similarly after many trials of practice, as in finger tapping speed (Peters, 1976), finger dexterity (Perelle et al., 1981; Bryden and Allard, 1998), finger movement sequencing (Teixeira and Okazaki, 2007), and older adults in pegboard tests (Weller and Latimer-Sayer, 1985). Our results differed from their results primarily in the almost identical performance of the two hands on Day 1, rather than an asymmetrical performance which might be expected in a sample of self-reported right handers. Our task also differed from theirs in several important ways. The first is that the task used in the present study is an isometric force control task, which involves no movement and minimal consequent central processing of movement-generated feedback (Lazarus and Haynes, 1997). The second is that our participants were screened on Day 1 for their understanding of the task by two joystick trials on each hand. The third difference is that unlike the tasks used in other studies cited, this isometric tracing task is not one encountered in activities of daily living in which the right hand may have experienced unequal amounts of practice. Therefore the two hands may have found the task equally as novel on the first day, but the more dominant right hand acquired the skill more quickly and plateaued.

### AGE AND TASK DIFFICULTY EFFECTS ON INTER-MANUAL PERFORMANCE ASYMMETRY

Task difficulty has been described as the level of complexity, (e.g., the portion of involved subsystems or abilities) that a task requires to complete it, or as “a skill that cannot be mastered in a single session, has more than one degree of freedom, and has the potential to be ecologically valid” (Wulf and Shea, 2002; Voelcker-Rehage, 2008, p. 64). The term *difficulty level* of a task has also been defined behaviorally by the time required to learn the task (Voelcker et al., 1999). In this study we presented a task requiring sustained applications and releases of isometric force, but introduced a change in difficulty in two locations along the tracing template, Segment 3 and Segment 6. The Segment main effect confirmed that these two segments took longer to navigate and generated larger mean error than the other four segments, confirming that within this task these two segments were more difficult for all age groups than the other four segments (**Figure 5**). Although the older groups tended to trace more slowly and make more errors, the age differences were not significant.

Inter-manual asymmetry were robustly different, however, beginning with Segment 3 and continuing through Segment 6 (**Figure 3**). The right hand (averaged across age groups) made fewer errors than the left hand on these last four segments. Thus, inter-manual asymmetry was more sensitive to changes in difficulty than the age factor was. Segment 3 is difficult because it requires the anticipation of a reversal of force direction (Spirduso et al., 2005b; Griffin et al., 2009; Eakin et al., 2012; Francis et al., 2012; Herring-Marler et al., 2014) approaching a target (Reverse Circle), requirements that are known to slow down the approach and induce errors (Salthouse, 1985). Segments 4 through 6 require the controlled release of force, known to be more difficult than controlled application of force which occurs in Segment 2 and Segment 3 (Spirduso and Choi, 1993).

Segment difficulty was also the most potent factor to affect the AI with regard to the time taken to complete the task (**Figure 6**). The difference between the performance of the hands was greater on Segment 3 and Segment 6, suggesting that the right hand completed the task more quickly than the left hand on the most difficult segments. It was not, however, more or less accurate than the left hand, indicating that a speed/accuracy tradeoff is not applicable.

In summary, old adults navigated the two most difficult segments as well as the young adults, as there were no interactions of age with segment or hand performance, either of inter-manual asymmetry or in the analysis of within-person AI. However, segment difficulty was a potent factor in the time taken by each hand to complete the task.

### LIMITATIONS

There were limitations in the present investigation. First, it focused on a specific motor task, isometric pinch force, and the findings more likely would be related to other studies of isometric force control rather than anisometric tasks. Second, the results of our study can only be generalized to an adult population who passed a stringent health screening, are well-educated and highly motivated. Therefore, the observed effects of aging, on manual asymmetry and isometric pinch force acquisition, likely reflect a best-case scenario of successful aging rather than a typical course of change.

## CONCLUSION

Results presented in this study show that when movement-generated feedback is absent or greatly reduced, as occurs in this isometric force control tasks, and when young and old participants are not time constrained, as occurs in tracing vs. tracking targets, age differences in inter-manual performance asymmetry are not found. These results do not support the HAROLD model but provide support for the notion that age and practice effects on asymmetry are task-specific. Practice effects depended on age level in that the Y20 and O70 groups improved performance by decreasing time taken to complete the task. However the O80 groups did not significantly improve with practice and practice did not significantly affect the AI for any age group. In addition, practice differentially affected the accuracy of performance of the two hands. Finally, all age groups exhibited poorer performance and larger AIs on the two most difficult segments (3 and 6) and this did not change with practice.

In conclusion, knowledge about isometric motor control in neurologically intact older adults is relevant to rehabilitation specialists such as physical therapists and occupational therapists who work with neuromuscularly impaired adults. Isometric tasks that can be performed by either hand and require very low levels of force are especially important tools for therapists working with patients attempting to recover from unilateral impairments in brain connectivity, such as stroke, fall-related concussions, or accidents.

## Conflict of Interest Statement

The authors declare that the research was conducted in the absence of any commercial or financial relationships that could be construed as a potential conflict of interest.
